# Association between Altered Oncogenic Signaling Pathways and Overall Survival of Patients with Metastatic Colorectal Cancer

**DOI:** 10.3390/diagnostics11122308

**Published:** 2021-12-08

**Authors:** Yi-Hsuan Huang, Peng-Chan Lin, Wu-Chou Su, Ren-Hao Chan, Po-Chuan Chen, Bo-Wen Lin, Meng-Ru Shen, Shang-Hung Chen, Yu-Min Yeh

**Affiliations:** 1 Department of Internal Medicine, National Cheng Kung University Hospital, College of Medicine, National Cheng Kung University, Tainan 70456, Taiwan; huangyh9184@gmail.com; 2 Department of Oncology, National Cheng Kung University Hospital, College of Medicine, National Cheng Kung University, Tainan 70456, Taiwan; pengchanlin@gmail.com (P.-C.L.); sunnysu@mail.ncku.edu.tw (W.-C.S.); 3 Division of Colorectal Surgery, Department of Surgery, National Cheng Kung University Hospital, College of Medicine, National Cheng Kung University, Tainan 70456, Taiwan; buoy2610@gmail.com (R.-H.C.); cpc324@gmail.com (P.-C.C.); wen276@gmail.com (B.-W.L.); 4 Department of Obstetrics and Gynecology, National Cheng Kung University Hospital, College of Medicine, National Cheng Kung University, Tainan 70456, Taiwan; mrshen@mail.ncku.edu.tw; 5 National Institute of Cancer Research, National Health Research Institutes, Tainan 70456, Taiwan

**Keywords:** metastatic colorectal cancer, next-generation sequencing, genomic profiling, oncogenic signaling pathways

## Abstract

Systemic characterization of genomic alterations into signaling pathways helps to understand the molecular pathogenies of colorectal cancer; however, their clinical implications remain unclear. Here, 128 patients with metastatic colorectal cancer (mCRC) receiving targeted next generation sequencing were retrospectively enrolled to analyze the impact of altered oncogenic pathways on clinical outcome. The datasets from Memorial Sloan Kettering Cancer Center were used for validation. In 123 patients with non-MSI-high tumor, the most common mutated gene was *TP53* (84.6%), followed by *APC* (78.0%), *KRAS* (49.6%), and *SMAD4* (22.8%). When mutated genes were allocated into signaling pathways defined as The Cancer Genome Atlas Pan-Cancer Analysis Project, alterations of cell cycle, Wnt, p53, RTK-RAS, PI3K, TGF-β, Notch, and Myc pathways were identified in 88%, 87%, 85%, 75%, 28%, 26%, 17%, and 10% of mCRC tissues, respectively. The survival analyses revealed that Myc and TGF-β pathway alterations were associated with a shorter overall survival (OS) (hazard ratio [HR]: 2.412; 95% confidence interval [CI]: 1.139–5.109; *p* = 0.018 and HR: 2.754; 95% CI: 1.044–7.265; *p* = 0.033, respectively). The negative prognostic impact of altered TGF-β pathway was maintained in patients receiving an anti-EGFR antibody. The OS of patients with mCRC carrying *MYC* and *BRAF* mutation was shorter than those with either *MYC* or *BRAF* mutation (HR: 4.981, 95% CI: 0.296–83.92; *p* = 0.02). These findings have clinical implications, such as prognosis prediction, treatment guidance, and molecular-targeted therapy development.

## 1. Introduction

Colorectal cancer is one of the most common cancer worldwide with approximately 1.2 million new cases annually [1]. Despite the introduction of early screening strategies, 35% of patients are diagnosed with distant metastasis at initial presentation [2]. In addition, 10–40% of patients experience disease metastasis after curative surgery [3]. Complete resection of metastatic lesions combined with systemic treatments provide prolonged survival and cure opportunity; however, metastatic colorectal cancer (mCRC) continued as a challenging public concern [4,5,6]. The 5-year survival rate of patients with mCRC remains less than 15% [2]. Therefore, a comprehensive understanding of molecular biology is important in the development of effective therapeutic strategies for mCRC.

CRC is a group of heterogenous diseases with diverse genomic features and corresponding treatment responses [7,8]. For example, anti-epidermal growth factor receptor (EGFR) antibodies combined with BRAF inhibitors are indicated for *BRAF*-mutated mCRC treatment; however, patients with mCRC harboring *RAS* mutation have a poor response to EGFR-targeting treatments [9,10,11]. With genetic and molecular analysis advancements, several studies revealed a comprehensive CRC genomic landscape and molecular profiles. The CRC Subtyping Consortium recognized a gene expression-based classification system that categorizes patients with CRC into four consensus molecular subtypes, including microsatellite instability (CMS1), canonical (CMS2), metabolic (CMS3), and mesenchymal (CMS4) to better integrate molecular features and clinical outcomes [12]. In a prospective study of targeted sequencing of 1134 mCRC tumor specimens, five subgroups for clinical implications are proposed based on mutations in the receptor tyrosine kinase (RTK), RAS-mitogen-activated protein kinase, or PI3K signaling pathway [13]. These genomic analyses provide deeper knowledge of complex molecular biology and the opportunity of the molecule-targeted therapeutic strategy of CRC.

A recent study by the framework of The Cancer Genome Atlas Research Network has mapped out genomic alterations in 10 canonical oncogenic pathways across 33 tumor types, including CRC [14]. Through this integrated genomic study, the intricate connection between various biological processes were distinguished in CRC progression. However, oncogenic pathway-associated clinical impact (e.g., treatment response and survival outcome) needs to be determined in mCRC. This present study aimed to evaluate the genomic alterations of oncogenic pathways and their clinical implications in Taiwanese patients with mCRC. The correlation between genomic profiles of canonical oncogenic pathways and parallel clinical outcomes were examined in the Taiwanese mCRC cohort, and compared with Memorial Sloan Kettering Cancer Center (MSKCC) dataset.

## 2. Materials and Methods

### 2.1. Study Cohort and Data Collection

Patients with mCRC receiving comprehensive genomic analyses at the NCKUH between January 2018 and May 2021 were enrolled. The clinicopathologic characteristics were collected from medical records. Mismatch repair status was determined using the immunohistochemical staining of MLH1, PMS2, MSH2, and MSH6. MSI status was analyzed using the polymerase chain reaction amplification of a set of mono- and dinucleotide repeats, including the BAT-25, BAT-26, NR-21, NR-24, and NR-27, followed by capillary electrophoresis and comparison of peak patterns. Right-sided colon cancer was defined as the primary tumor arising in the cecum, ascending colon, hepatic flexure, and transverse colon, whereas primary tumors located in the splenic flexure, descending colon, sigmoid colon, and rectum was defined as left-sided colon cancer. Comprehensive genomic profiling of tumor tissues was determined using the hybridization capture-based NGS (the FoundationOne CDx assay) [15]. Tumor tissue manipulation, genomic DNA extraction, library preparation, sequencing, and data analyses were carried out as previously described [16,17]. TMB and MSI results were simultaneously profiled. All types of genetic alterations, including substitutions, small insertion and deletions (Indels), copy number alterations, and gene rearrangements, were examined in pathway analyses. The alterations classified as variants of unknown significance were excluded. This study was approved by the institutional review board of the NCKUH (protocol code: B-ER-110-172, date of approval: 23 June 2021) and conducted following the Declaration of Helsinki.

### 2.2. Analysis of Altered Signaling Pathways

All examined genetic alterations were assigned into 10 signaling pathways defined as The Cancer Genome Atlas Pan-Cancer Analysis Project, including RTK-RAS, PI3K, β-catenin/Wnt, Myc, TGF-β, Notch, p53, cell cycle, Hippo, and oxidative stress response/Nrf2 pathway [14]. The alteration frequencies for each gene in pathway diagrams were examined using PathwayMapper [18], a web-based visualization tool. The OncoPrint graphs, which reveal the co-occurrence of various genomic alterations in the signaling pathway, were generated using the online tool of the cBioportal website [19]. Alteration of signaling pathways was defined as the identification of one or more genomic alterations associated with the pathway.

### 2.3. Comparison of Datasets from NCKUH and MSKCC Cohort

The genomic profiling of patients with mCRC using a capture-based targeted NGS assay was reappraised from the MSKCC dataset for comparison studies [13]. The clinical characteristics of patients with CRC from the MSKCC cohort, including the age, gender, primary tumor location, tumor sample used for sequencing, and MSI status, were obtained from the cBioportal website [19]. All types of alterations, including the missense mutation, inframe mutation, splice mutation, truncating mutations, structural variants, amplification, and deletion, were queried using the cBioportal interface for alteration frequency determination.

### 2.4. Statistical Analysis

A chi-square test or the Fisher exact test was used to determine the associations of variables, depending on the group size. An unpaired t-test or Mann Whitney U test was used to compare the mean of each group based on the nature of the variable. The OS was defined as the time from the initial mCRC diagnosis to death from any cause. The Kaplan-Meier method with a log-rank test was applied for survival analyses. The association between the OS and genetic alterations was assessed by the Cox proportional hazards regression model. The genetic alterations present in at least 5 patients were indicated for multivariable regression analysis. All the statistical analyses were conducted using the R statistical software version 4.0.5. A *p* value of <0.05 was considered statistically significant.

## 3. Results

### 3.1. Characteristics of NCKUH Cohort

A total of 128 patients with mCRC receiving targeted-next-generation sequencing (NGS)-based genomic profiling at National Cheng Kung University Hospital (NCKUH) were included. The clinical characteristics of these patients were summarized in Table 1. Among 128 patients, 111 (86.7%) patients received 5-fluorouracil plus irinotecan (FOLFIRI) with or without a targeted agent, such as an anti-EGFR antibody (cetuximab and panitumumab) and anti-vascular endothelial growth factor (VEGF) antibody (bevacizumab) as first-line treatment. Seven (5.5%) patients received 5-fluorouracil plus oxaliplatin in combination with a target agent as the initial treatment.

The Microsatellite instability (MSI) status analysis was examined in all 128 patients using the PCR-based technique; the MSI status using the NGS-based analyses was evaluated in 108 patients. Patients were divided into subgroups based on MSI status by PCR-based technique, wherein 5 and 123 patients were categorized in MSI-high (MSI-H) and non-MSI-H CRC groups, respectively. MSI statuses determined by PCR and NGS-based techniques were highly concordant in this cohort. Among 108 patients with NGS-determined MSI status, 4 and 104 patients remained with MSI-H and non-MSI-H phenotypes according to PCR-based approach, respectively. The age of patients with MSI-H CRC was significantly younger than those with non-MSI-H CRC (47 vs. 61 years old; *p* = 0.02; Table 1). The percentage of right-sided CRC in MSI-H group was higher compared to that of the non-MSI-H group (80.0% vs. 20.0%; *p* = 0.0018; Table 1).

**Figure 1:**
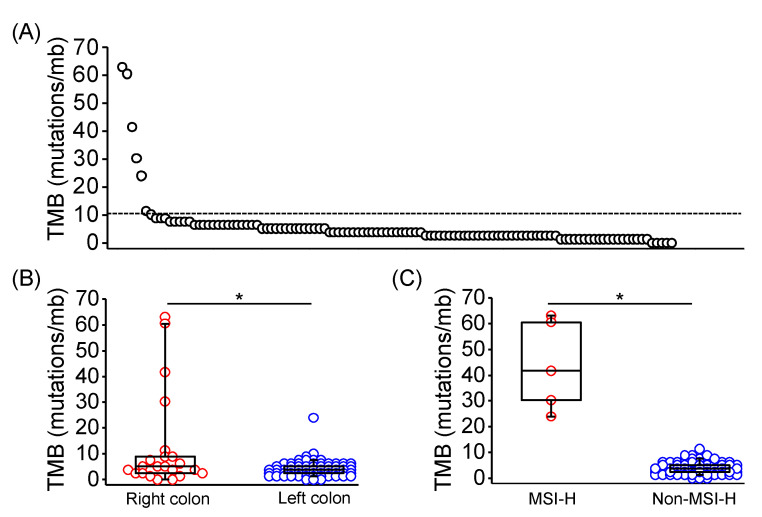
Tumor mutation burden in Taiwanese patients with mCRC. (**A**) TMB in each of 113 evaluated tumor tissues using the targeted-NGS technique. The dashed line indicated 10 mutations/mb. TMB in mCRC with distinct primary location (**B**), and MSI-H presentation (**C**) was shown and compared using the Mann Whitney U test. The horizontal line denoted the median, the upper and lower bottom of the box indicated the 25th and 75th percentile, and the whiskers marked the 5th and 95th percentiles. * represented *p* < 0.05.

Tumor mutation burden (TMB) is an emerging biomarker for immune checkpoint inhibitor response across several tumor types, including CRC [20,21]. Through calculation from genomic profiles of each sample, TMB was evaluated in 113 of 128 patients, and 7 of 113 samples exhibited TMB high tumors (TMB > 10 mutations/mb; Figure 1A). TMB of right-sided CRC were significantly higher than left-sided CRC (median TMB: 4.41 vs. 2.52 mutations/mb; *p* = 0.0348; Figure 1B). Moreover, TMB of MSI-H CRC was significantly higher compared with non-MSI-H CRC (median TMB: 41.61 vs. 3.78 mutations/Mb; *p* < 0.0001; Figure 1C). *POLE* mutation was identified in one MSI-H CRC sample with a TMB of 60.5 mutations/mb.

### 3.2. Genomic Alterations in Non-MSI-H CRC

**Figure 2:**
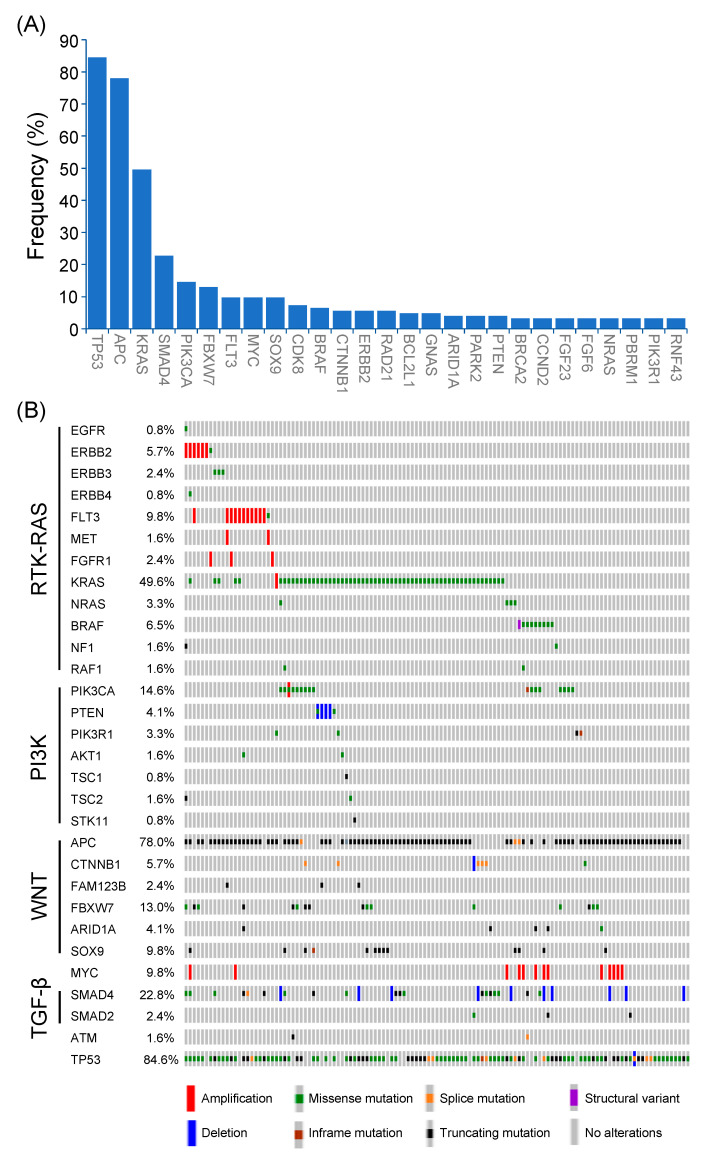
Genetic alterations of Taiwanese patients with non-MSI-H mCRC. (**A**) Frequencies of genetic alterations (>3%) were identified in non-MSI-H mCRC. (**B**) The frequencies, types, and co-occurrences of mutated genes in major oncogenic pathways.

Among 123 patients with non-MSI-H mCRC, a total of 457 genetic alterations involved in 98 genes were identified (Figure 2A). The most common mutated gene was *TP53* (84.6%), followed by *APC* (78.0%), *KRAS* (49.6%), *SMAD4* (22.8%), *PIK3CA* (14.6%), *FBXW7* (13.0%), *FLT3* (9.8%), *MYC* (9.8%), *SOX9* (9.8%), *CDK8* (7.3%), and *BRAF* (6.5%). The types and co-occurrence of genomic alterations were presented in Figure 2B.

The genomic profiles from the MSKCC dataset were reappraised to compare the prevalence of genomic alterations between the Western and Eastern patients with mCRC [13]. As shown in Figure S1A, the percentage of patients aged ≥ 60 years was significantly higher in the NCKUH cohort compared with the MSKCC dataset (49.2% vs. 36.2%; *p* = 0.004). The percentage of right-sided (22.7% vs. 30.1%; *p* = 0.0675) and MSI-H CRC (3.9% vs. 8.7%; *p* = 0.0620) was lower in the NCKUH cohort compared with the MSKCC dataset. Among the commonly mutated genes, the alteration frequencies of *APC*, *KRAS*, *FBXW7*, *SMAD4*, *PIK3CA*, *SOX9*, *FLT3*, and *BRAF* were similar between the NCKUH and MSKCC cohort, whereas mutation frequencies of *TP53* and *MYC* were higher in the NCKUH cohort (Figure S1B).

The genomic alteration-associated prognosis was further evaluated in patients with mCRC. By using Cox proportional hazards regression model, results revealed that the overall survival (OS) of patients with *BRAF*, *ARID1A*, *MYC*, and *SMAD4* mutations was significantly shorter than wild-type population (Table S1). The mutated genes identified in at least 5 patients were referred to as multivariate analyses. As shown in Table S1, mutated *BRAF* was the only independent prognostic factor associated with a worse OS in patients with mCRC (Hazard ratio [HR] for death: 6.1423; 95% confidence interval [CI]: 1.9784–19.0694; adjusted *p* = 0.0371).

### 3.3. Alterations of Signaling Pathways

**Figure 3:**
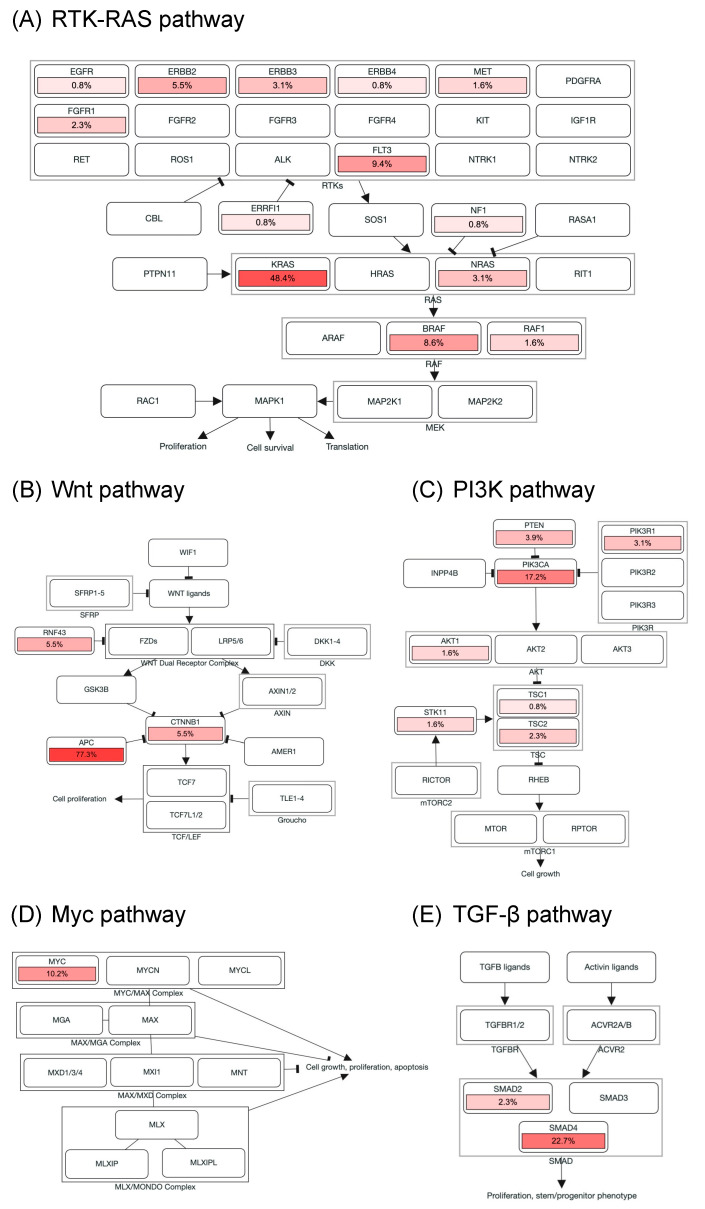
Alterations of signaling pathways in Taiwanese mCRC. Genomic alterations of the RTK-RAS (**A**), Wnt (**B**), PI3K (**C**), Myc (**D**), and TGF-β pathway (**E**). The color intensity represented the alteration frequency of pathway members. An arrow indicated an activation; a bar at the end of an edge indicated an inhibitory interaction.

Genomic alterations were assigned to 10 signaling pathways as defined in previous studies to investigate the correlation between oncogenic pathways and survival outcome in patients with mCRC [14,18]. As shown in Figure 3 and Figure S2, cell cycle alterations, Wnt, p53, RTK-RAS, PI3K, TGF-β, Notch, and Myc pathway were observed in 88%, 87%, 85%, 75%, 28%, 26%, 17%, and 10% of mCRC tissues, respectively; no genetic alteration was detected in Hippo and Nrf2 pathway. The major genes associated with the RTK-RAS pathway included *KRAS* (48.4%), *FLT3* (9.4%), *BRAF* (8.6%), *ERBB2* (5.5%), *NRAS* (3.1%), *ERBB3* (3.1%), and *FGFR1* (2.3%; Figure 2B and Figure 3A). The most commonly mutated gene observed in the Wnt pathway was *APC* (77.3%), followed by *CTNNB1* (5.5%) and *RNF43* (5.5%; Figure 2B and Figure 3B). Among key genes in the PI3K pathway, *PIK3CA*, *PETN*, *PIK3R1*, and *TSC2* mutations were identified in 17.2%, 3.9%, 3.1%, and 2.3% of mCRC tissues, respectively (Figure 2B and Figure 3C). Regarding genomic alterations of the Myc pathway, *MYC* was the only altered gene and identified in 10.2% of CRC tissues (Figure 3D). A TGF-β signaling pathway is involved in CRC progression through invasion and metastasis promotion of cancer cells [8]. In the present study, the TGF-β pathway alteration frequency was 26%, including *SMAD4* and *SMAD2* mutations in 22.7% and 2.3% samples, respectively (Figure 2B and Figure 3E). The cell cycle and p53 pathway were the upmost signaling pathways with frequent alterations identified in the NCKUH cohort (88% and 85%, respectively). The main mutated gene in both two pathways was *TP53* (82.8%; Figure S2).

### 3.4. Clinical Outcome of Signaling Pathway with Alteration

**Figure 4:**
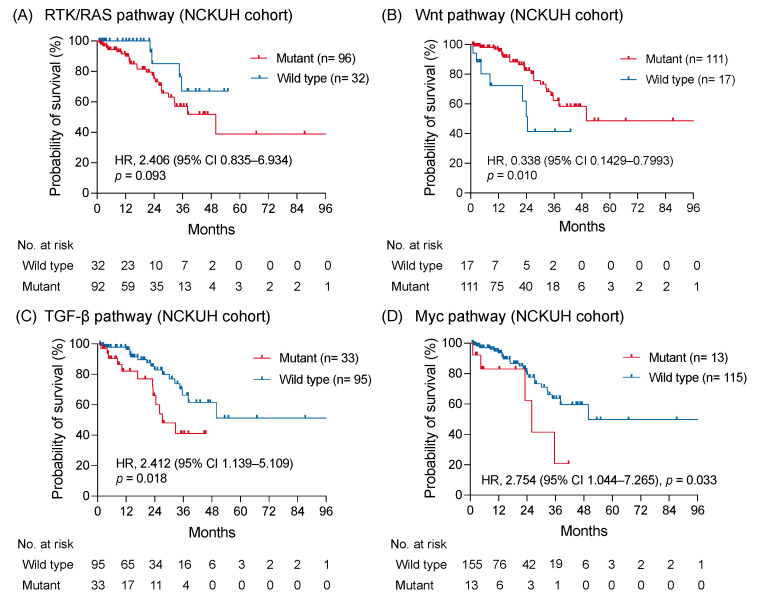
The survival impact of altered signaling pathways. The Kaplan-Meier curves of overall survival in patients with mCRC with and without altered RTK-RAS (**A**), Wnt (**B**), TGF-β (**C**), and Myc pathway (**D**) were shown and compared using a log-rank test.

In the NCKUH cohort, the median OS was 49.9 months without a significant difference between right-sided and left-sided mCRC (*p* = 0.42; Figure S3A,B). By using the Kaplan-Meier method, a trend showing a worse OS was identified in patients with RTK-RAS pathway alterations compared with patients without RTK-RAS pathway alterations (HR: 2.406; 95% CI: 0.835–6.934; *p* = 0.093; Figure 4A). The OS of patients with Wnt pathway alterations was longer than those without alterations (HR: 0.338; 95% CI: 0.1429–0.7993; *p* = 0.010; Figure 4B). Notably, the altered TGF-β and Myc pathways were significantly associated with a shorter OS (HR: 2.412; 95% CI: 1.139–5.109; *p* = 0.018 and HR: 2.754; 95% CI: 1.044–7.265; *p* = 0.033, respectively; Figure 4C,D). No significant survival difference was observed between patients with and without genomic alterations of PI3K, Notch, cell cycle, and p53 pathway (Figure S3C–F). The correlation between the survival outcomes and investigated pathways from the MSKCC dataset was examined to validate the prognostic impact of genomic-altered signaling on mCRC [11]. Comparable to findings from the NCKUH cohort, the altered TGF-β pathway correlated with a shorter OS, whereas the Wnt pathway correlated with a longer OS in the MSKCC cohort (Figure S4).

**Figure 5:**
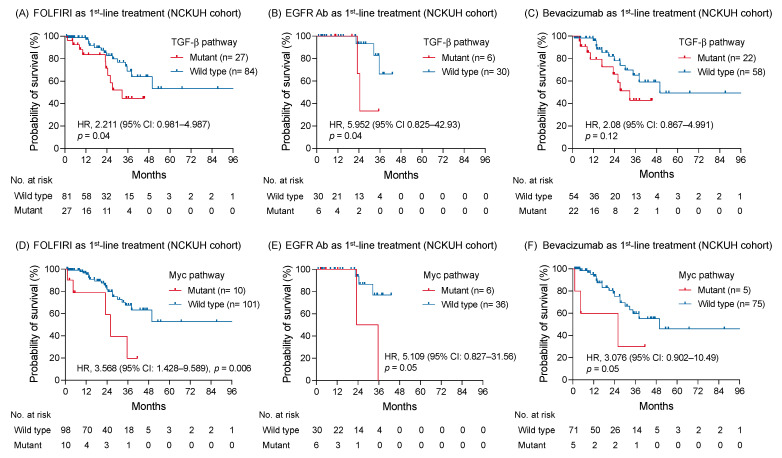
The correlation between altered signaling pathways and survival of patients with mCRC receiving disparate first-line treatment. The Kaplan-Meier survival curves of patients with and without genomic alterations of TGF-β pathway receiving FOLFIRI regimen (**A**), an anti-EGFR antibody (**B**), and bevacizumab (**C**) as first-line treatment. The association between the altered Myc pathway and survivals of patients receiving FOLFIRI (**D**), an anti-EGFR antibody (**E**), and bevacizumab (**F**) as first-line treatment were compared using a log-rank test, as well as the altered TGF-β pathway.

The correlation was further examined between the oncogenic pathway alteration and survival outcome of a specific treatment type. In patients receiving FOLFIRI regimen as first-line treatment, the OS with genomic alterations of TGF-β pathway remained shorter than those without alterations (HR: 2.211; 95% CI: 0.981–4.987; *p* = 0.04; Figure 5A). An anti-EGFR or VEGF antibody was concomitantly used with chemotherapy as first-line treatment for 36 (28.1%) and 80 (62.5%) patients, respectively. Among patients receiving an anti-EGFR antibody, genomic alterations of the TGF-β pathway correlated with a worse OS, compared to those without alterations (HR: 5.952; 95% CI: 0.825–42.93; *p* = 0.04; Figure 5B). In contrast, no survival difference was found between tumors with and without TGF-β pathway alterations when bevacizumab was used as first-line treatment (HR: 2.08; 95% CI: 0.867–4.991; *p* = 0.12; Figure 5C). Regarding patients with *RAS* wild-type mCRC receiving bevacizumab as first-line treatment, survival outcomes were also similar between groups with and without TGF-β pathway alterations (Figure S5A).

The altered Myc pathway maintained its negative prognostic effect on patients receiving FOLFIRI regimen as first-line treatment (HR: 3.568; 95% CI: 1.428–9.589; *p* = 0.006; Figure 5D). A trend appealing a shorter OS was found in patients with tumor carrying Myc pathway alterations compared to those without alterations whether an anti-EGFR or VEGF antibody was used as first-line treatment (HR: 5.109; 95% CI: 0.827–31.56; *p* = 0.05 and HR: 3.076; 95% CI: 0.902–10.49; *p* = 0.05, respectively; Figure 5E,F). Among 13 patients with *MYC* mutation, 4 (30%) patients simultaneously harbored *BRAF V600E* mutation. Patients with either *MYC* or *BRAF* mutations were associated with a worse OS compared with those without mutation (Figure S5B,C). Moreover, patients with *MYC* and *BRAF* mutation co-occurrence had a shorter OS than those with either *MYC* or *BRAF* mutation (HR: 9.772; 95%CI: 0.8739–109.3; *p* = 0.02; Figure S5D).

## 4. Discussion

CRC is a group of heterogenous diseases exhibiting a wide range of genomic features and corresponding clinical presentations. The comprehensive genomic profiling from 128 Taiwanese patients with mCRC was identified in this study. Genomic features of Taiwanese mCRC were generally similar to the Western dataset from the MSKCC cohort. The survival-related oncogenic pathway analyses revealed that inferior survivals were associated with altered TGF-β and Myc pathway and superior survivals are associated with altered Wnt pathway. These results provide practical information about mCRC management, including treatment preference, prognosis prediction, and therapeutic development.

The TGF-β signaling pathway regulates tissue development and homeostasis, and associated genomic alterations are involved in CRC progression [12,13,14,22,23,24]. Genomic TGF-β pathway alterations were identified in 26% Taiwanese patients with mCRC compared to previous studies. Several reports suggest that TGF-β is a prognostic marker for early-stage CRC [12,24,25]. The NCKUH and MSKCC dataset revealed that altered TGF-β pathway was associated with a worse OS of patients with mCRC. Notably, TGF-β pathway alterations correlated with a shorter OS in patients receiving an anti-EGFR antibody as first-line treatment, but not those treated with an anti-VEGF antibody. Anti-EGFR therapy is important in the treatment of mCRC, thus several molecular mechanisms of therapy resistance are investigated, mainly focusing on the RTK-RAS pathway [26,27,28,29]. Some pre-clinical studies demonstrated that the TGF-β pathway is related to anti-EGFR therapy resistance through *AKT* activation or *SMAD4*-associated epithelial-mesenchymal transition [30,31]. Several therapies targeting the TGF-β pathway are clinically developing, efficacy evaluation of these novel targeted therapies combined with anti-EGFR therapy is encouraged in patients with mCRC [32,33,34]. Collectively, our findings support the role of the TGF-β pathway playing in the pathogenesis of mCRC and resistance mechanism to anti-EGFR therapy.

The transcription factor Myc regulates numerous genes involved in cellular functions, such as cell growth, metabolism, and apoptosis [35,36,37]. In this study, genomic alterations of the Myc pathway, all of which were *MYC* amplification, were identified in 13 of 128 Taiwanese patients with mCRC. Several investigators examined the clinical impact of Myc expression on CRC; however, this evidence remains inconclusive [38,39,40,41,42]. The prognostic impact discordance between these studies is related to distinct analytic tools in Myc expression detection (e.g., immunohistochemistry and in situ hybridization), patients with dissimilar disease stages, and limited sample size. Importantly, the present study is the first one to reveal the negative prognostic impact of *MYC* amplification in patients with mCRC based on the targeted-NGS platform. Furthermore, the survival trend remained inferior in patients with *MYC*-amplified mCRC receiving chemotherapy combined with anti-EGFR or anti-VEGF therapy. Accordingly, the correlation between the altered Myc pathway and shorter OS may infer attenuated chemotherapy response in patients with *MYC*-amplified mCRC. Indeed, the most commonly-used chemotherapy drugs for mCRC are almost DNA-damaging agents [43,44]. *MYC* expression is reported to induce activation of genes that control DNA synthesis and regulate chemotherapy drug resistance [45,46,47,48]. The powerful chemotherapy regimen with triplet combination (i.e., oxaliplatin, irinotecan, and 5-fluorouracil) are indicated in patients with *MYC*-amplified mCRC and a feasible physical condition to overcome *MYC*-related drug resistance.

The co-occurrence of *BRAF V600E* mutation was found in 4 of 13 *MYC* mutated mCRC to extend the Myc pathway interrogation. Several clinical studies revealed that *BRAF V600E* mutation is a poor prognostic factor of mCRC [49,50], including our multivariate survival analyses in Taiwanese patients. The current treatments of *BRAF*-mutated mCRC include a triplet combination chemotherapy regimen and the combination of anti-BRAF and EGFR therapy [51,52]. However, diverse responses to systemic treatments among patients with *BRAF*-mutated mCRC indicate the presence of genomic heterogeneity in this aggressive CRC subtype [52,53]. In this study, the survival of patients with *BRAF* and *MYC* mutation co-occurrence was shorter than those with either *BRAF* or *MYC* mutation. Moreover, some in vitro studies demonstrated that *MYC* is a key element in tumor progression and treatment resistance of *BRAF*-mutated CRC [54,55]. Studies investigating effective treatments are warranted for this aggressive subtype of CRC carrying both the *BRAF* and *MYC* mutation.

Clinical characteristics and genomic profiles of 128 Taiwanese patients with mCRC are comprehensively collected and systemically analyzed; a relatively small sample size is the major limitation of this study. Additionally, selection bias is present due to the retrospective approach of this study. Therefore, genomic profiles from the MSKCC dataset were applied to validate the prognostic value of the TGF-β and Wnt pathway in mCRC. Furthermore, alterations in a total of 324 genes were detected using the targeted-NGS platform, and a few pathway-associated genomic alterations with a low mutation frequency were unidentified in this study. Importantly, these bioinformatic analyses findings may lead basic studies with precise designs to explore the convoluted biological network composed by signaling pathways in CRC.

## 5. Conclusions

In summary, genomic alterations of TGF-β and Myc pathway correlated with inferior survivals of patients with mCRC. Furthermore, the negative prognostic impact of altered TGF-β pathway in patients receiving anti-EGFR therapy guides the treatment preference. Co-occurrence of *BRAF* and *MYC* mutation in mCRC tissues confer the worst prognosis, and treatments of this aggressive type of CRC are prudent. Our studies provide several novel insights into molecular mechanisms of CRC progression and the development of effective therapeutic strategies against mCRC.

## Figures and Tables

**Table 1:** Clinical characteristics of the 128 mCRC patients.

Characteristic	All Subjects	MSI-H	Non-MSI-H
Total patients	128	5	123
Age—yr			
Median	61	47	61
Range	26–87	37–66	26–87
Gender—no. (%)			
Male	76 (59.4)	4 (80.0)	72 (58.5)
Female	52 (40.6)	1 (20.0)	51 (41.5)
Primary tumor location—no. (%)			
Right-sided	29 (22.7)	4 (80.0)	25 (20.3)
Left-sided	99 (77.3)	1 (20.0)	98 (79.7)
Tumor sample for testing—no. (%)			
Primary lesion	71 (55.5)	3 (60.0)	68 (55.3)
Metastatic lesion	57 (44.5)	2 (40.0)	55 (44.7)
Tumor histology—no. (%)			
Adenocarcinoma	122 (95.3)	5 (100)	117 (95.1)
Mucinous adenocarcinoma	6 (4.7)	0	6 (4.9)
Tumor grade—no. (%)			
Well differentiated	5 (3.9)	0	5 (4.1)
Moderately differentiated	102 (79.7)	4 (80.0)	98 (79.7)
Poorly differentiated	12 (9.4)	1 (20.0)	11 (8.9)
NA	9 (7.0)	0	9 (7.3)
First-line regimen—no. (%)			
FOLFIRI + Bevacizumab	75 (58.6)	3 (60.0)	72 (58.5)
FOLFIRI + Cetuximab	18 (14.1)	0	18 (14.6)
FOLFIRI + Panitumumab	14 (10.9)	0	14 (11.4)
FOLFIRI	4 (3.1)	0	4 (3.3)
FOLFOX + Bevacizumab	3 (2.3)	1 (20.0)	2 (1.6)
FOLFOX + Cetuximab	1 (0.8)	0	1 (0.8)
FOLFOX + Panitumumab	3 (2.3)	0	3 (2.4)
FOLFOXIRI + Bevacizumab	2 (1.6)	0	2 (1.6)
FOLFOXIRI	1 (0.8)	1 (20.0)	0
Capecitabine or UFUR	4 (3.1)	0	4 (3.3)
Palliative RT alone	1 (0.8)	0	1 (0.8)
Cetuximab + Dabrafenib + Trametinib	2 (1.6)	0	2 (1.6)

Abbreviations: MSI-H, microsatellite instability-high; FOLFIRI, 5-fluorouracil, folinic acid, irinotecan; FOLFOX, 5-fluorouracil, folinic acid, oxaliplatin; FOLFOXIRI, 5-fluorouracil, folinic acid, irinotecan, oxaliplatin; NA, not available; RT, radiotherapy.

## Data Availability

Not applicable.

## Supplementary Materials

The following are available online at https://www.mdpi.com/article/10.3390/diagnostics11122308/s1, Figure S1: Comparison of the dataset from MSKCC and NCKUH; Figure S2: Genetic alterations of the Notch, cell cycle, and p53 pathways in Taiwanese patients with mCRC; Figure S3: The survival impact of tumor sidedness and altered signaling pathways in the NCKUH cohort; Figure S4: The survival impact of altered TGF-β and Wnt pathway in the MSKCC cohort; Figure S5: The survival impact of co-occurrence of *MYC* and *BRAF* mutation; Table S1: The association between mutated genes and overall survival of patients with metastatic colorectal cancer.
