# Supplementary material for: Association between Altered Oncogenic Signaling Pathways and Overall Survival of Patients with Metastatic Colorectal Cancer
Source: Diagnostics (Basel). 2021 Dec 8;11(12):2308. doi: 10.3390/diagnostics11122308 (PMC8700603; doi:10.3390/diagnostics11122308)

Supplemental figures

Figure S1. Comparison of the dataset from MSKCC and NCKUH. (**A**) Comparison of the clinical characteristics between the MSKCC and NCKUH cohorts. (**B**) Comparison of alteration frequencies of 16 major mutated genes in the MSKCC and NCKUH cohorts.

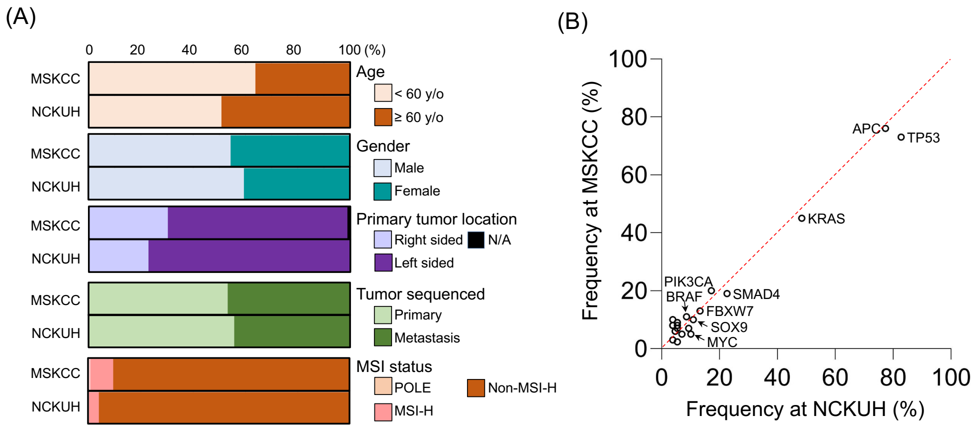

Figure S2. Genetic alterations of the Notch (**A**), cell cycle (**B**), and p53 pathways (**C**) in Taiwanese patients with mCRC.

(A) Notch pathway

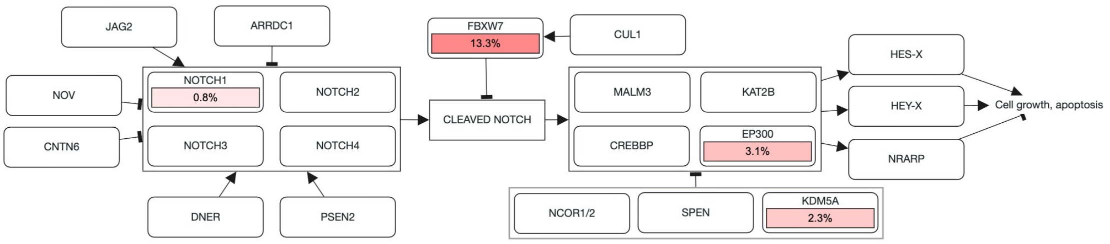

(B) Cell cycle pathway

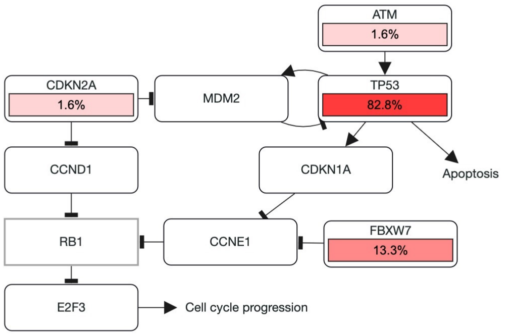

(C) p53 pathway

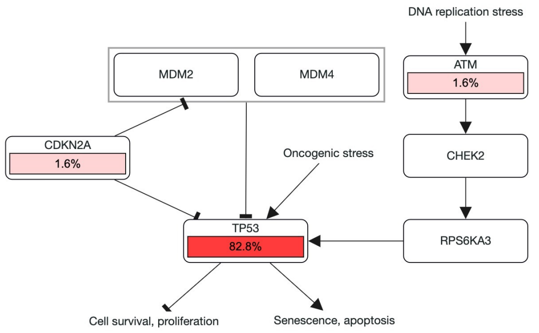

Figure S3. The survival impact of tumor sidedness and altered signaling pathways in the NCKUH cohort. (**A**) The Kaplan-Meier curves of 128 Taiwanese patients with mCRC. The correlations between the overall survival and tumor sidedness (**B**), the presence of altered PI3K (**C**), Notch (**D**), cell cycle (**E**), and p53 pathway (**F**) were analyzed using a log-rank test.

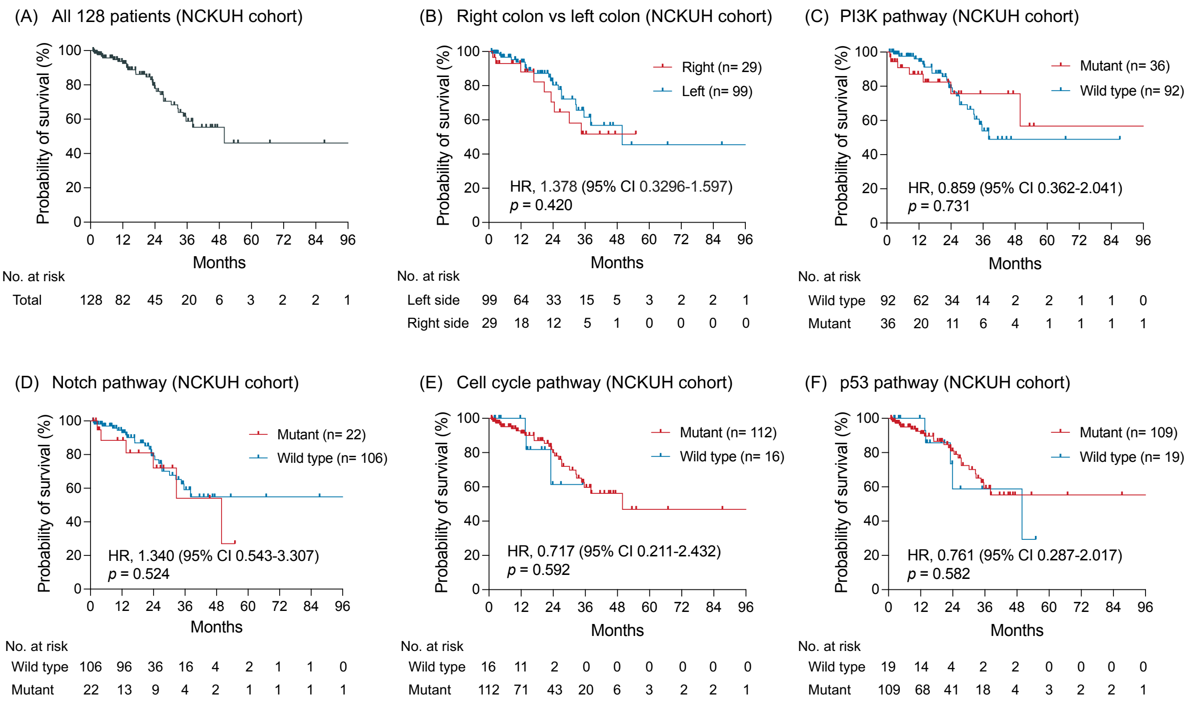

Figure S4. The survival impact of altered TGF-β and Wnt pathway in the MSKCC cohort. The Kaplan-Meier curves of patients collected in the MSKCC cohort were shown. The correlations between the overall survival and presence of altered TGF-β (**A**) and Wnt pathway (**B**) were analyzed using a log-rank test. The red line indicated the altered group and the blue line indicated the wild-type population.

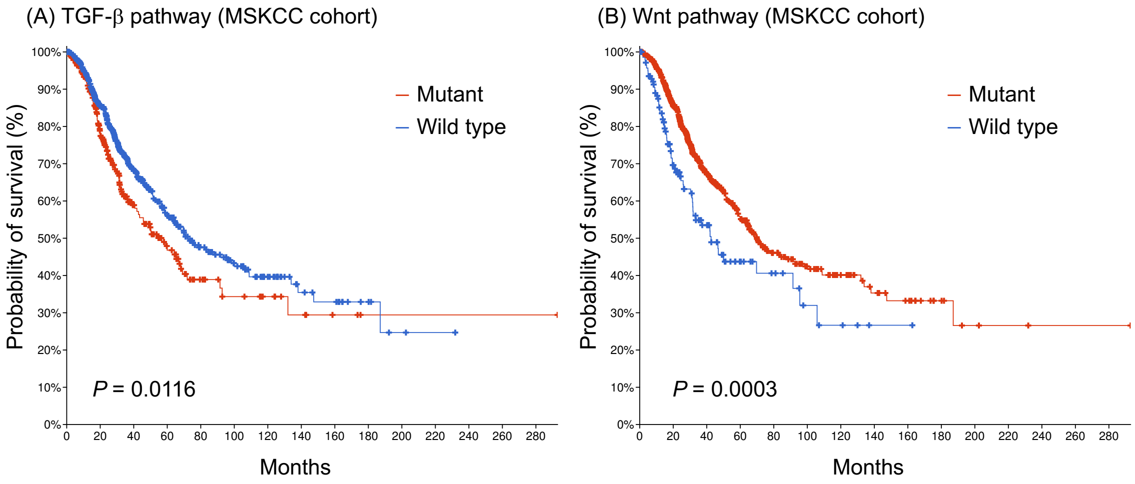

Figure S5. The survival impact of co-occurrence of MYC and BRAF mutation. (**A**) The Kaplan-Meier curves of Taiwanese patients with *RAS* wild-type mCRC receiving bevacizumab as first-line treatment was shown. The correlation between the overall survival and altered TGF-β pathway was performed using a log-rank test. The Kaplan-Meier curves of patients with and without *MYC* (**B**) and *BRAF* mutation (**C**) were shown and their associate prognoses were compared using a log-rank test. (**D**) The comparison of the overall survival between patients with co-occurrence of *MYC* and *BRAF* mutation and those with either *MYC* and *BRAF* mutation was shown.

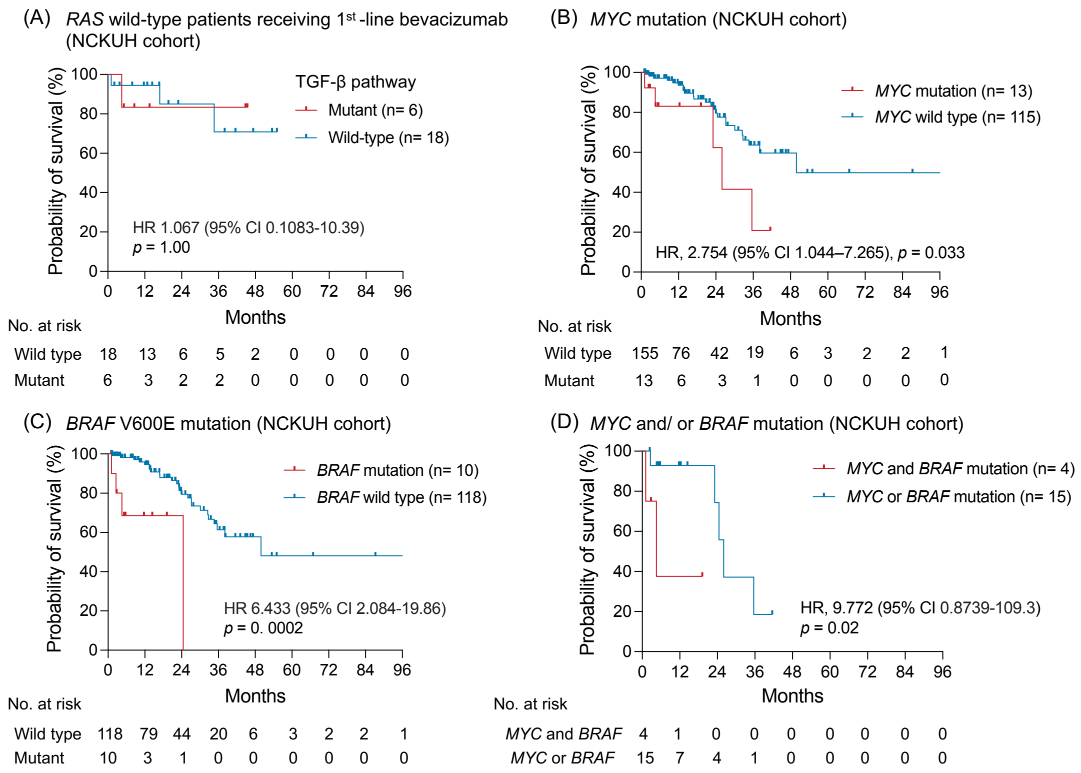

Supplement: Supplementary file 1 [file diagnostics-11-02308-s001.zip › Supplementary figures_211204.pdf]
