# Supplementary material for: Association between Altered Oncogenic Signaling Pathways and Overall Survival of Patients with Metastatic Colorectal Cancer
Source: Diagnostics (Basel). 2021 Dec 8;11(12):2308. doi: 10.3390/diagnostics11122308 (PMC8700603; doi:10.3390/diagnostics11122308)
Supplement: Supplementary file 1 [file diagnostics-11-02308-s001.zip › Supplemental table_diagnostics.pdf]

Supplemental table

Table S1. The association between mutated genes and overall survival of patients with metastatic colorectal cancer.

Gene name	Case number with mutation	Case number without mutation	*P* value	Adjusted *P* value	Hazard ratio	95% confidence interval	
						Lower limit	Upper limit
BRAF	11	117	0.001687	0.037114006	6.142332404	1.978468151	19.06942366
ARID1A	7	121	0.024043014	0.243919795	4.034160619	1.201132241	13.54925907
MYC	13	115	0.04061589	0.243919795	2.75435251	1.044233394	7.265097814
SMAD4	29	99	0.044349054	0.243919795	2.21497427	1.02029915	4.808502502
KRAS	62	66	0.090461381	0.398030077	1.941807937	0.900632288	4.186634337
APC	99	29	0.316822782	0.834980188	0.673798336	0.311023577	1.459709907
BCL2L1	6	122	0.418888873	0.834980188	1.642607046	0.493106488	5.471755034
FLT3	12	116	0.419542519	0.834980188	0.552706776	0.130995874	2.332018339
PIK3CA	22	106	0.454328596	0.834980188	1.439867918	0.554108823	3.74153873
FBXW7	17	111	0.498159819	0.834980188	0.66029787	0.198688514	2.19435572
SOX9	14	114	0.552246051	0.834980188	1.378413774	0.478398831	3.971632893

Gene name	Case number with mutation	Case number without mutation	*P* value	Adjusted *P* value	Hazard ratio	95% confidence interval	
						Lower limit	Upper limit
RAD21	7	121	0.581839612	0.834980188	1.499196164	0.354789295	6.334997043
BRCA2	5	123	0.583040594	0.834980188	1.502328367	0.351272764	6.425179376
ERBB2	7	121	0.610938508	0.834980188	0.594976897	0.080488312	4.398123158
TP53	106	22	0.629022551	0.834980188	0.786389169	0.296644127	2.084679482
PARK2	5	123	0.633973567	0.834980188	1.420460995	0.334947907	6.023949978
PTEN	5	123	0.676342533	0.834980188	0.652731926	0.088088288	4.836726636
MLL2	5	123	0.705379276	0.834980188	0.678715681	0.091019383	5.061064568
GNAS	6	122	0.721119253	0.834980188	1.301138321	0.306628332	5.521214952
CTNNB1	7	121	0.83896059	0.922856649	1.132471245	0.341167674	3.759122617
CDK8	9	119	0.974416881	0.997347057	0.976724098	0.231560175	4.119836078
RNF43	7	121	0.997347057	0.997347057	1.23E-08	0.000000000	Inf
